# Interrelation of Individual, Country and Activity Constraints in Motor Activities of Daily Living among Typically Developing Children: A Cross-sectional Comparison of Spanish and Dutch Populations

**DOI:** 10.3390/ijerph17051705

**Published:** 2020-03-05

**Authors:** Laura Delgado-Lobete, Rebeca Montes-Montes, Sonia Pértega-Díaz, Sergio Santos-del-Riego, José-Manuel Cruz-Valiño, Marina M. Schoemaker

**Affiliations:** 1 Faculty of Health Sciences, Health Integration and Promotion Research Unit (INTEGRA SAÚDE), University of A Coruña, 15011 A Coruña, Spain; sergio.santos.delriego@udc.es (S.S.-d.-R.); director@iciro.es (J.-M.C.-V.); 2 Center for Human Movement Sciences, University Medical Center Groningen, University of Groningen, 9713 AV Groningen, The Netherlands; m.m.schoemaker@umcg.nl; 3 Faculty of Health Sciences, TALIONIS Research Group, University of A Coruña, 15008 A Coruña, Spain; 4 Institute of Biomedical Research of A Coruña (INIBIC), University Hospital Complex A Coruña (CHUAC), Faculty of Health Sciences, University of A Coruña, 15006 A Coruña, Spain; sonia.pertega.diaz@sergas.es

**Keywords:** developmental coordination disorder, dynamic systems theory, cross-cultural, motor performance, activities of daily living, occupational therapy, DCDDaily-Q

## Abstract

Motor performance is influenced by individual, environmental, and task constraints. Children perform differently according to individual (i.e., sex), environmental (i.e., country), and task (i.e., type of activity) factors. However, little is known about the effect of the interaction between sex and country factors across different activities of daily living (ADL) learning, participation, and performance. The main aim of this study was to examine the relationship between sex, country, and type of activity in motor-based ADL learning, participation, and performance in five-to-eight-year-old, typically developing children. Additionally, we aimed to compare the prevalence of probable Developmental Coordination Disorder (DCD) across sex and country. The DCDDaily-Q was used to assess ADL learning, participation, and performance in 300 age and sex-matched children from Spain and The Netherlands. The prevalence of probable DCD was determined based on the total ADL performance score. Results showed that differences in ADL learning, participation and performance differed across sex and country (*p* < 0.05). Prevalence of probable DCD was statistically similar in both countries. These findings show that daily participation and performance in typically developing children may be influenced by individual, country, and task constraints, and that country and sex may have different influences on particular tasks.

## 1. Introduction

The Dynamic Systems Theory proposes that motor performance results from the interaction of individual, environmental, and task constraints [1,2,3]. Previous research has proposed that both motor performance and the presence of Developmental Coordination Disorder (DCD) are influenced by different constraints such as sex, lifestyle, physical activity routines, environmental settings, and participation in activities of daily living (ADL) [4,5,6,7,8,9]. The influence of these factors varies from one country to another, even within a Western European context, and there is an increasing interest in literature to explore motor performance patterns in children from different regions [4,6,10,11,12,13].

Previous studies investigating geographical or country influences using the Movement Assessment Battery for Children-Second Edition (MABC-2) have found significant differences in motor competence between regions. Findings indicate that Brazilian children perform significantly poorer on manual dexterity and balance than American children [10], while Czech children outperform children from the United Kingdom (UK) in the same domains [11].

Apart from geographical constraints, also individual constraints such as age and sex may influence motor performance even within the same country. While the performance of 3–6-year-old Dutch children was similar to children in the UK, Dutch children older than 6 showed better outcomes on manual dexterity, aiming and catching-ball skills, and balance on the MABC-2 [12]. In some studies, individual constraints such as age were found to interact with country constraints. For instance, Zoia et al. found that younger Italian children obtained lower scores than children in the UK on manual dexterity, balance and aim, and catching-ball skills, while this difference was overturned when children get older, as older Italian children generally performed better than British children in all components [13].

Additionally, the influence of sex as an individual constraint on motor coordination has been repeatedly reported in previous studies, but the results are inconclusive, as not every study has found differences between boys and girls. This inconsistency could be due to the different methods used to assess motor competence (i.e., objective motor tests such as the MABC-2 or parental questionnaires such as the Developmental Coordination Disorder Questionnaire), since it has been reported that boys tend to outperform girls in gross motor-based evaluations while girls are more proficient in fine motor skills and control during movement skills, as handwriting and balance [8,11].

Sex differences in motor performance can also be influenced by country context, as children’s everyday participation is still sex-biased, and boys and girls are often encouraged to engage in different physical and leisure activities [14,15]. As a consequence, motor performance across sex can present differently in different countries. Psotta et al. found that Czech girls showed better manual dexterity than UK girls, despite boys’ performance is similar in both countries [11]. Brazilian girls are less proficient in manual dexterity than American girls, while Brazilian boys score significantly poorer on ball skills [10]. Children with DCD struggle with a broad range of daily motor activities and especially with self-care activities [7], but little is known about if and how country differences influence participation and performance in self-care ADL.

Country constraints may also have an effect on the performance of motor Activities of Daily Living (ADL). Motor performance during ADL is usually assessed with parent questionnaires, being the Developmental Coordination Disorder Questionnaire (DCDQ), the most often used measure. Caravale et al. found that parents rated the motor performance of Italian children lower than parents of Canadian children with the DCDQ, suggesting poorer daily motor performance in Italian children [16]. Conversely, parents rated the ADL performance of five-to-eight-year-old German children as significantly better than parents of Canadian children [17]. In both cases, specific cutoff scores for Italian and German children were developed in order to assure a correct evaluation of motor performance in these populations.

When motor coordination difficulties have a significant and constant impact on the performance of daily living activities (i.e., limiting self-care activities performance and participation), then the child may be at risk for DCD [18]. The DCD is a chronic condition with lifelong consequences in physical and psychosocial health, participation restriction, and academic achievement [19,20,21,22,23,24]. The American Psychiatric Association estimates that this disorder affects approximately 6% of school-aged children [18], but different rates have been reported for children of European, American, and Latin American regions, suggesting an influence of country factors on prevalence rate. Southern European and Latin American children usually showed a higher prevalence of DCD or probable DCD than Northern European or American children [8,25,26,27,28], but few studies have directly compared the prevalence of DCD in two or more populations from different countries.

Tsiotra et al. found that Greek children demonstrate higher DCD prevalence rates when compared to Canadian children despite both samples coming from Western countries, suggesting a direct influence of differences in lifestyle on the prevalence of DCD [6]. More recently, Valentini et al. reported that Brazilian children were twice more likely to show probable DCD than American children [10]. In both studies, the prevalence of DCD was established with objective motor coordination evaluation (the Bruininks–Oseretsky Test of Motor Proficiency and the MACB-2, respectively). It should be noted that children were classified as having DCD using original cutoffs and not country-adjusted cutoffs, which could partially explain these outcomes. These studies demonstrate that cultural background associate with DCD rates across regions.

Overall, there seems to be a significant difference in both motor performance and prevalence of DCD between children from Southern and Northern regions, both in America and within Europe. However, no studies exist that further explore the influence of sex (individual constraint) on learning, participation, and performance of motor-based ADL across countries (environmental constraint) and type of activity (task constraint). Further research regarding the interrelation of these factors is needed to understand how individual, environmental, and task constraints may associate with daily performance and participation in typically developing children. Therefore, the aims of this study are:

- To explore country differences between children from a Northern European country (The Netherlands) and a Southern European country (Spain) in learning, participation, and performance of motor-based ADL as evaluated by the DCDDaily-Q.
- To examine the relationship between sex and country and learning, participation and performance of ADL as evaluated by the DCDDaily-Q.
- To explore country differences in the prevalence of probable DCD between Dutch and Spanish children as operationalized by the DCDDaily-Q.

## 2. Materials and Methods

### 2.1. Study Design, Participants and Procedure

We conducted a multicenter cross-sectional study in Spain and The Netherlands. This study was approved by the Autonomic Research Ethics Committee of Galicia, Spain (code 2018-606). The Dutch part of the study was approved by the Medical Ethics Committee of the University Medical Centre Groningen. All participants consented to take part in the study anonymously and confidentially.

The sample comprised two subgroups of 150 Spanish and 150 Dutch five-to-eight-year-old typically developing children matched by exact age and sex. This sample size was estimated in order to measure the effect of country on ADL performance (effect size d = 0.389, α = 0.05, power (1 − β) = 0.90) [29,30]. Spanish children were randomly selected from a larger sample from ten randomly selected schools in four different regions in northwest, north, and central Spain between January and December 2019 [30]. The Dutch group was randomly matched by age and sex from a previously recruited reference sample of Dutch children from different regions of the Netherlands [29]. Children were excluded beforehand if they had a parent-reported or clinically diagnosed neurodevelopmental disorder, such as DCD, Attention Deficit Hyperactivity Disorder, or Autism Spectrum Disorder. In both samples, parents anonymously completed the DCDDaily-Q within a week and then returned it to the researchers.

### 2.2. Outcome Measures

The Dutch and Spanish versions of the DCDDaily-Q were used to assess ADL learning, participation, and performance. The DCDDaily-Q is a 23-item parent questionnaire that evaluates a broad range of ADL in children aged five-to-eight years old, including self-care, fine motor, and gross motor activities [29]. Parents are asked to state how frequently their child perform each activity (1 = regularly, 2 = sometimes, 3 = seldom, 4 = not yet/never, total score = 23 to 92), their proficiency while doing so (1 = good, 2 = medium, 3 = poor, total score = 23 to 69) and whether their child took longer to learn the activity than other children (0 = no, 1 = yes, total score = 0 to 23). Higher scores on participation and performance subscales reflect less participation and poorer motor performance, respectively, while total learning scores show how many ADL the child took longer to learn compared to their peers. The total ADL and subscales scores (i.e., self-care, fine motor, and gross motor ADL) are calculated for learning, participation and performance. Based on learning total and subscale scores, children can be classified as “typical learning” if they score 0 or “took longer to learn at least one ADL” if they score ≥ 1. Additionally, the child can be identified as having probable DCD according to the total ADL performance score; the child can be identified as having probable DCD. An example of the items of the DCDDaily-Q is provided in Appendix Table A1. The DCDDaily-Q was originally developed and validated in Dutch children, showing excellent psychometric properties and capacity to discriminate children with DCD from typically developing children (Cronbach alpha = 0.85; sensitivity = 88%; specificity = 92%) [29].

This questionnaire has recently been cross-culturally adapted and validated in the Spanish population, and its three-dimensional structure, reliability, and validity have been confirmed (Cronbach alfa = 0.86) (to be published). Country-adjusted reference norms have also been developed for Spanish children aged 5 to 10 years old [30]. Based on the total ADL performance score, a child has probable DCD if they have a total score ≥ 95th percentile of their age group (Dutch criteria = age 5 ≥ 43; age 6 ≥ 40; ages 7 and 8 ≥ 37; Spanish criteria = ages 5 and 6 ≥ 45; ages 7 to 10 ≥ 39) [29,30].

### 2.3. Data Analysis

Analyses were performed using the IBM Statistical Package for Social Sciences Version 25 (SPSS Inc., Chicago, IL, USA). The sample size was estimated using G*Power version 3.1.9.4. (Heinrich-Heine-Universität Düsseldorf, Düsseldorf, Germany) [31]. Data were examined for normality using visual inspection and skewness and kurtosis [32]. Differences in participation and performance according to country and sex were assessed with independent *t*-tests and multivariate analyses. Differences in the prevalence of delayed learning of ADL were calculated using Chi-square tests.

First, differences were explored between Spanish and Dutch children and boys and girls independently. Further analyses were conducted to determine differences in learning, participation, and performance in Spanish and Dutch children according to sex.

Next, linear regression models using a stepwise method were performed to explore the interrelation between sex and country on those ADLs, which were independently and differently influenced by sex and country during bivariate analysis. Assumptions of normality, linearity, homoscedasticity, and absence of multicollinearity were tested.

Finally, differences in the prevalence of probable DCD between Spanish and Dutch children and boys and girls were calculated using chi square tests.

## 3. Results

Sample characteristics are displayed in Appendix Table A2. The sample was balanced by age, sex and country (*n* = 300; 5 years old = 24.0%, 6 years old = 25.3%, 7 years old = 25.3%, 8 years old = 25.3%; boys in each age group = 50.0%; Spanish = 50.0%).

### 3.1. The Interrelation of Sex, Country, and Activity on Motor Performance and Daily Participation

#### 3.1.1. Differences in ADL Learning

Bivariate analysis showed that there were no significant differences in the time it took to learn self-care, fine motor, gross motor, or total ADL between Spanish and Dutch children as reported by parents (Table 1). Parents in the combined sample reported that boys took longer to learn self-care, fine motor and total ADL (Table 1).

However, some differences in learning were found between countries, as parents reported that Dutch boys took overall longer to learn fine motor, and total ADL, but these differences were not reported by Spanish parents (Table 2). Finally, learning of ADL was similar between Spanish and Dutch children when analyzing boys and girls separately (Table 3).

#### 3.1.2. Differences in ADL Participation

Parents of Dutch children reported more participation in self-care activities in their offspring than parents of Spanish children (Table 1). Boys in the overall sample were reported to participate less than girls in self-care, fine motor and total ADL. Further analysis across each country and sex groups showed that both Spanish and Dutch boys participated less than girls in fine motor ADL, but some differences were present (Table 2). Dutch boys participated less than Dutch girls in total ADL, but Spanish boys participated less than Spanish girls in self-care activities according to parents. When analyzing boys and girls separately, both Spanish boys and girls participated less in self-care ADL than Dutch boys and girls, although differences were higher and more significant in boys (Table 3).

#### 3.1.3. Differences in ADL Performance

Finally, differences in performance according to country and sex were analyzed. Dutch children performed better than Spanish children in self-care, gross motor, and total ADL as reported by parents (Table 1). Parents in the overall sample reported that girls performed better than boys in self-care, fine motor and total ADL (Table 1).

Both Spanish and Dutch boys performed worse than Spanish and Dutch girls in fine motor ADL, but Spanish boys also performed worse than Spanish girls in self-care activities, according to parents, while Dutch boys and girls performed equally in self-care activities (Table 2).

Differences in the performance of ADL between Spanish and Dutch subsamples emerged across sex as well (Table 3). Spanish boys performed worse than Dutch boys in self-care and total ADL as rated by parents. Although Spanish girls also participated less in self-care ADL, their performance in self-care ADL was similar to Dutch girls’ performance. However, parents rated Spanish girls to perform significantly poorer than parents of Dutch girls in gross motor ADL, even though the gross motor performance was rated similarly for Spanish and Dutch boys.

Overall, discrepancies in findings for between sex, country, and type of activity were present for total ADL performance, self-care participation and self-care performance, and therefore, three linear regression models were conducted with these three factors as dependent variables, and sex and country as predictors (Table 4). The three models met the assumptions of normality, linearity, homoscedasticity, and absence of multicollinearity. The analysis showed that both country and sex significantly predicted total ADL performance and self-care ADL participation and performance. However, the country showed a greater effect on participation in self-care ADL than sex.

### 3.2. Prevalence of probable Developmental Coordination Disorder

Prevalence of probable DCD was statistically similar in Spanish and Dutch groups (Spanish = 8.0%, Dutch = 6.7%, *p* = 0.658). Prevalence of probable DCD was almost twice in boys compared to girls, but this difference was not significant (boys = 9.3%, girls = 5.3%, *p* = 0.184). This higher but non-significant difference between boys and girls was also present when analyzing across countries (prevalence in Spanish children: boys = 10.7%, girls = 5.3%, *p* = 0.229; prevalence in Dutch children: boys = 8.0%, girls = 5.3%, *p* = 0.513).

## 4. Discussion

The main objective of this study was to explore sex and country differences in motor-based ADL learning, participation, and performance in five-to-eight-year-old, typically developing children from two countries of South and North Europe.

Preliminary bivariate analyses highlighted country and sex differences in self-care, fine motor, gross motor and total ADL learning, participation, and performance. Further analyses determined that differences in ADL learning, participation, and performance diverged across sex and countries, especially in relation to total ADL performance and self-care ADL participation and performance.

Results from this study contribute to explain disagreements found in previous research regarding motor competence and sex. The two main instruments used to assess motor proficiency and performance are the MABC-2 and the DCDQ, which involve balance/control during movement, fine, and gross motor activities [5,33,34]. While there seems to be an agreement regarding girls outperforming boys in fine motor activities, results concerning a sex gap in other areas or in general coordination are often inconclusive. Previous research has already argued that girls get fewer opportunities to practice gross motor activities, encouragement and reinforcement, while simultaneously participating more in drawing and cutting activities, resulting in different motor competence patterns [35]. Consequently, it is to be expected to find that, in general, children show more proficiency in those activities which they engage in and practice more frequently (i.e., fine motor and self-care activities for girls, and gross motor and dynamic activities for boys).

These findings also suggest a country’s influence on differences in motor performance patterns across sex, especially in self-care activities. The sex gap in participation in self-care and household chores has been consistently and repeatedly reported in previous studies carried out in different countries, socioeconomic, and cultural backgrounds [36,37,38,39]. Interestingly, in this study, the sex gap was only present in the Spanish group, as Dutch boys and girls showed similar participation and performance in self-care ADL. This is consistent with previous studies showing different patterns across sex in participation in self-care and house chores between children from northern and southern Europe [36,37]. For instance, Giménez-Nadal et al. found that the sex gap in self and house care participation is greater in Spanish children in comparison with German children [37]. While these studies focused on children older than eight years, the present work suggests that a sex gap in ADL participation may be present before the age of eight and will likely persist as children grow older [36].

Our findings contribute to support a complex relationship between the influence of environmental, activity and sex factors on daily participation and performance, as country and sex had a similar effect over total ADL and self-care ADL motor performance, but country played a more relevant role in participation in self-care ADL. This situation has not been explored before but has relevant implications for the understanding of the underlying factors of both participation and performance in daily living in typically developing children. As those two aspects are correlated in both samples, it can be assumed that Spanish girls but not Dutch girls outperforming boys in self-care activities points to sex stereotyping related factors, as girls are encouraged to participate more in self-care activities, instead of actual sex differences in motor capacity. In conclusion, these outcomes link to the Dynamic System Theory and suggest an even further interrelationship between individual (i.e., sex), environmental (i.e., country and participation differences), and task (i.e., type of activity) constraints regarding motor performance.

Despite self-care activities being one of the main occupational areas of interest in childhood, they are rarely part of motor assessment tools, and consequently, relevant information is often lacking in studies regarding the daily impact of DCD. Self-care and instrumental ADL are two of the area’s that children with DCD and other neurodevelopmental conditions most struggle with [7,40,41,42]. Therefore, assessment protocols of motor coordination in daily living aimed to identify children at risk for DCD should systematically include self-care and instrumental ADL evaluation.

Regarding DCD prevalence, boys were twice as likely as having probable DCD than girls, but this difference was not significant. This was also observed when the country was considered, as both Spanish girls and Dutch girls showed a higher but not significantly different prevalence of probable DCD than boys. Overall, these findings are consistent with two recent studies in Spanish preschoolers and school-aged children that found similar rates of probable DCD using the DCDQ and the MABC-2 [8,25]. Thus, the inclusion of self-care activities in the assessment of motor performance and risk for DCD makes for a more comprehensive evaluation without misrepresenting DCD prevalence in the population.

Although differences in probable DCD between Spanish and Dutch children were not significant, a higher prevalence of DCD in southern European children has been persistently reported [6,8,12,25,26,28,39,43,44,45,46]. It is to be expected to find higher prevalence in regions with lower performance scores on DCD assessment measures, but a higher percentage of children with probable DCD in southern European regions is present even in those countries with population-adjusted cutoff scores, like Italy or Spain [25,26]. It should be noted that the prevalence of DCD can be influenced by the instrument used to determine the diagnosis, as objective motor coordination assessment through motor tests may differ from parent evaluation. However, high probable DCD rates have been reported in south Europe regardless of the type of instrument used [8,25].

One country-related factor in explaining these findings could be the existing differences in physical activity rates between European regions, as low participation in physical activities has been previously associated with risk for DCD and poor moto competence [6,8,47,48]. However, the results of studies investigating differences in participation in physical activity across European countries are inconclusive [47]. This situation adds to the evidence of motor behavior and coordination difficulties resulting from the dynamic interrelation of different factors, as individual and geographical constraints determine motor learning and practice opportunities.

Overall, this study has several implications for clinical practice and research. Health and rehabilitation practitioners, such as pediatric occupational therapists, can use these findings to further promote performance through participation in children with motor coordination issues, which is in compliance with the International Classification of Functioning, Disability, and Health [49]. These outcomes further support the Dynamic Systems Theory, which has relevant implications for research practice and can contribute to understanding the underlying mechanisms of motor performance both in typically developing children and in children with DCD.

The current study is subject to certain limitations and recommendations for future research directions. This was a cross-sectional study, and therefore, causal relationships cannot be estimated. The Dutch subsample came from the DCDDaily-Q standardization study in The Netherlands, which could influence the prevalence of probable DCD in Dutch children. Efforts have been made to obtain a representative and balanced sample to try to avoid further biases and support the generalizability of the results (i.e., the sample comes from different geographical locations in each country, is age and country balanced, and there is an equal sex distribution across every age group and country). Additionally, findings obtained from parental questionnaires should be interpreted cautiously, as parents’ perceptions are subjective. Nonetheless, parents can provide reliable and valuable information about their child’s everyday performance, which is difficult to determine in a clinical evaluation [29]. Additional lifestyle- and play-related variables were not collected, which may affect internal validity, and therefore further research should explore potential confounding effects. Finally, the diagnosis of definite DCD could not be determined as only one diagnostic criterion was assessed (i.e., criterion B = daily motor performance). In order to minimize the risk of false-positive classification, the 95th and not the 85th percentile cutoff score was used to determine probable DCD in the sample, as it is recommended when addressing the presence of DCD in population-based research studies [50,51].

## 5. Conclusions

This study indicates that daily participation and performance in typically developing children are associated with individual, task, and country constraints and that this relationship is dynamic and varies between contexts. This is the first study to explore the influence of the interrelation between sex, type of activity and geographical background on ADL learning, participation, and performance. These findings may have relevant implications for both the clinical field and research, especially in relation to the role of Dynamic Systems Theory in motor performance. The sex gap in learning, participation, and performance of motor-based daily activities seems to rely on geographical background and type of activity, showing an even more complex interrelation between individual, environmental, and task constraints. It is necessary to develop population-adjusted cutoff scores to prevent a cultural bias when addressing the diagnosis of DCD in different countries, regardless of the instrument of evaluation.

## Figures and Tables

**Table 1:** Learning, participation, and performance of ADL according to country and sex (*n* = 300).

DCDDaily-Q Subscales	Spanish *n* = 150	Dutch *n* = 150	*p* Value	Boys *n* = 150	Girls *n* = 150	*p* Value
	N (%)	N (%)		N (%)	N (%)	
Delayed learning of ADL						
Self-care ADL	22 (14.7)	18 (12.0)	0.497	27 (18.0)	13 (8.7)	0.017
Fine motor ADL	17 (11.3)	14 (9.3)	0.569	23 (15.3)	8 (5.3)	0.004
Gross motor ADL	16 (10.7)	8 (5.3)	0.089	11 (7.3)	13 (8.7)	0.670
Total ADL	38 (25.3)	28 (18.7)	0.163	42 (28.0)	24 (16.0)	0.012
	**Mean (SD)**	**Mean (SD)**		**Mean (SD)**	**Mean (SD)**	
Participation of ADL						
Self-care ADL	15.4 (3.8)	13.9 (3.2)	0.000	15.2 (3.8)	14.1 (3.3)	0.011
Fine motor ADL	9.4 (2.4)	9.7 (2.2)	0.433	9.9 (2.4)	9.2 (2.1)	0.006
Gross motor ADL	12.1 (2.9)	11.9 (2.9)	0.561	12.1 (2.8)	12.0 (2.9)	0.888
Total ADL	37.0 (7.4)	35.5 (6.2)	0.072	37.2 (6.8)	35.3 (6.8)	0.022
Performance of ADL						
Self-care ADL	13.9 (3.2)	12.9 (2.4)	0.004	13.8 (2.9)	13.0 (2.8)	0.017
Fine motor ADL	9.2 (2.3)	9.2 (2.1)	0.958	9.8 (2.3)	8.6 (1.9)	<0.001
Gross motor ADL	9.5 (2.5)	8.7 (2.3)	0.006	9.0 (2.3)	9.2 (2.6)	0.495
Total ADL	32.5 (6.7)	30.8 (5.5)	0.015	32.6 (6.0)	30.8 (6.2)	0.012

ADL = activities of daily living; SD = standard deviation.

**Table 2:** Differences in learning, participation and performance of ADL in boys and girls according to country (*n* = 300).

	Spanish Subsample			Dutch Subsample		
DCDDaily-Q Subscales	Boys *n* = 75	Girls *n* = 75	*p* Value	Boys *n* = 75	Girls *n* = 75	*p* Value
	N (%)	N (%)		N (%)	N (%)	
Delayed learning of ADL						
Self-care ADL	15 (20.0)	7 (9.3)	0.065	12 (16.0)	6 (8.0)	0.132
Fine motor ADL	11 (14.7)	6 (8.0)	0.198	12 (16.0)	2 (2.7)	0.005
Gross motor ADL	7 (9.3)	9 (12.0)	0.597	4 (5.3)	4 (5.3)	1.000
Total ADL	22 (29.3)	16 (21.3)	0.260	20 (26.7)	8 (10.7)	0.012
	**Mean (SD)**	**Mean (SD)**		**Mean (SD)**	**Mean (SD)**	
Participation of ADL						
Self-care ADL	16.1 (4.0)	14.7 (3.5)	0.021	14.3 (3.3)	13.6 (3.0)	0.197
Fine motor ADL	9.6 (2.4)	9.3 (2.3)	0.561	10.3 (2.3)	9.0 (1.8)	<0.001
Gross motor ADL	12.0 (3.0)	12.3 (2.8)	0.551	12.1 (2.7)	11.8 (3.1)	0.430
Total ADL	37.6 (7.6)	36.3 (7.2)	0.261	36.7 (6.0)	34.4 (6.3)	0.025
Performance of ADL						
Self-care ADL	14.4 (3.3)	13.3 (3.1)	0.027	13.1 (2.4)	12.7 (2.4)	0.278
Fine motor ADL	9.8 (2.4)	8.6 (1.9)	0.001	9.8 (2.2)	8.6 (1.9)	0.001
Gross motor ADL	9.3 (2.4)	9.7 (2.6)	0.398	8.7 (2.0)	8.7 (2.6)	0.917
Total ADL	33.5 (6.8)	31.5 (6.5)	0.065	31.6 (5.1)	30.1 (5.8)	0.086

ADL = activities of daily living; SD = standard deviation.

**Table 3:** Differences in learning, participation, and performance of ADL in Spanish and Dutch children according to sex (*n* = 300).

	Boys			Girls		
DCDDaily-Q Subscales	Spanish *n* = 75	Dutch *n* = 75	*p* Value	Spanish *n* = 75	Dutch *n* = 75	*p* Value
	N (%)	N (%)		N (%)	N (%)	
Delayed learning of ADL						
Self-care ADL	15 (20.0)	12 (16.0)	0.524	7 (9.3)	6 (8.0)	0.772
Fine motor ADL	11 (14.7)	12 (16.0)	0.821	6 (8.0)	2 (2.7)	0.146
Gross motor ADL	7 (9.3)	4 (5.3)	0.347	9 (12.0)	4 (5.3)	0.147
Total ADL	22 (29.3)	20 (26.7)	0.716	16 (21.3)	8 (10.7)	0.075
	**Mean (SD)**	**Mean (SD)**		**Mean (SD)**	**Mean (SD)**	
Participation of ADL						
Self-care ADL	16.1 (4.0)	14.3 (3.3)	0.003	14.7 (3.5)	13.6 (3.0)	0.045
Fine motor ADL	9.6 (2.4)	10.3 (2.3)	0.073	9.3 (2.3)	9.0 (1.8)	0.391
Gross motor ADL	12.0 (3.0)	12.1 (2.7)	0.773	12.3 (2.8)	11.8 (3.1)	0.280
Total ADL	37.6 (7.6)	36.7 (6.0)	0.385	36.3 (7.2)	34.4 (6.3)	0.090
Performance of ADL						
Self-care ADL	14.4 (3.3)	13.1 (2.4)	0.006	13.3 (3.1)	12.7 (2.4)	0.208
Fine motor ADL	9.8 (2.4)	9.8 (2.2)	0.917	8.6 (1.9)	8.6 (1.9)	0.966
Gross motor ADL	9.3 (2.4)	8.7 (2.0)	0.096	9.7 (2.6)	8.7 (2.6)	0.032
Total ADL	33.5 (6.8)	31.6 (5.1)	0.046	31.5 (6.5)	30.1 (5.8)	0.144

ADL = activities of daily living; SD = standard deviation.

**Table 4:** Linear regression models for total ADL performance, self-care ADL participation and self-care ADL performance (stepwise method) (*n* = 300).

**Dependent variable: Total ADL performance**				
Predictors	B	95% Confidence interval		*p* Value
		Lower limit	Upper limit	
Sex	1.773	0.396	3.150	0.012
Country	1.720	0.343	3.097	0.015
**Dependent variable: Self-Care ADL participation**				
Predictors	B	95% Confidence interval		*p* Value
		Lower limit	Upper limit	
Sex	1.040	0.256	1.824	0.009
Country	1.440	0.656	2.224	< 0.001
**Dependent variable: Self-care ADL performance**				
Predictors	B	95% Confidence interval		*p* Value
		Lower limit	Upper limit	
Sex	0.793	0.154	1.433	0.015
Country	0.940	0.300	1.580	0.004

ADL = activities of daily living; B = B coefficient value.

## Appendix A

**Table A1:** Illustrative item of the DCDDaily-Q.

**Item 1. Activity: ** Buttering a sandwich	**Correct performance:** The right amount of butter is neatly and evenly spread, at a normal pace, without making a mess and without dangerous situations involving the knife	
**Participation** *My child does this…* 1. Regularly 2. Sometimes 3. Seldom 4. Not yet / never	**Performance** *My child can do this…* 1. Well 2. Sometimes well and sometimes not as well 3. Not very well (or badly) most of the time	**Learning** *My child…* 1. Is taking or has taken longer to learn this skill than his/her age peers

**Table A2:** Sociodemographic characteristics of the sample (*n* = 300).

Sociodemographic Characteristics	Spanish	Dutch
	N (%)	N (%)
5 years old	36 (24.0)	36 (24.0)
Boys (5 years old)	18 (50.0)	18 (50.0)
Girls (5 years old)	18 (50.0)	18 (50.0)
6 years old	38 (25.3)	38 (25.3)
Boys (6 years old)	19 (50.0)	19 (50.0)
Girls (6 years old)	19 (50.0)	19 (50.0)
7 years old	38 (25.3)	38 (25.3)
Boys (7 years old)	19 (50.0)	19 (50.0)
Girls (7 years old)	19 (50.0)	19 (50.0)
8 years old	38 (25.3)	38 (25.3)
Boys (8 years old)	19 (50.0)	19 (50.0)
Girls (8 years old)	19 (50.0)	19 (50.0)
Total	150 (50.0)	150 (50.0)
